# Engineering the Surface and Mechanical Properties of Water Desalination Membranes Using Ultralong Carbon Nanotubes

**DOI:** 10.3390/membranes8040106

**Published:** 2018-11-13

**Authors:** Yehia M. Manawi, Kui Wang, Viktor Kochkodan, Daniel J. Johnson, Muataz A. Atieh, Marwan K. Khraisheh

**Affiliations:** 1 Qatar Environment and Energy Research Institute (QEERI), Hamad Bin Khalifa University (HBKU), Qatar Foundation, Doha, Qatar; yehmanawi@hbku.edu.qa (Y.M.M.); vkochkodan@hbku.edu.qa (V.K.); mhussien@hbku.edu.qa (M.A.A.); 2 School of Traffic and Transportation Engineering, Central South University, Changsha 410075, China; kui.wang@outlook.com; 3 Centre for Water Advanced Technologies and Environmental Research (CWATER), College of Engineering, Swansea University, Swansea SA2 8PP, UK; djj9000@googlemail.com; 4 College of Science and Engineering, Hamad Bin Khalifa University, Qatar Foundation, Doha, Qatar

**Keywords:** membranes, atomic force microscopy, mechanical properties, water desalination

## Abstract

In this work, novel polysulphone (PS) porous membranes for water desalination, incorporated with commercial and produced carbon nanotubes (CNT), were fabricated and analyzed. It was demonstrated that changing the main characteristics of CNT (e.g., loading in the dope solutions, aspect ratio, and functionality) significantly affected the membrane properties and performance including porosity, water flux, and mechanical and surface properties. The water flux of the fabricated membranes increased considerably (up to 20 times) along with the increase in CNT loading. Conversely, yield stress and Young’s modulus of the membranes dropped with the increase in the CNT loading mainly due to porosity increase. It was shown that the elongation at fracture for PS/0.25 wt. % CNT membrane was much higher than for pristine PS membrane due to enhanced compatibility of commercial CNTs with PS matrix. More pronounced effect on membrane’s mechanical properties was observed due to compatibility of CNTs with PS matrix when compared to other factors (i.e., changes in the CNT aspect ratio). The water contact angle for PS membranes incorporated with commercial CNT sharply decreased from 73° to 53° (membrane hydrophilization) for membranes with 0.1 and 1.0 wt. % of CNTs, while for the same loading of produced CNTs the water contact angles for the membrane samples increased from 66° to 72°. The obtained results show that complex interplay of various factors such as: loading of CNT in the dope solutions, aspect ratio, and functionality of CNT. These features can be used to engineer membranes with desired properties and performance.

## 1. Introduction

Global fresh water scarcity and pollution is becoming one of the most critical issues due to rapid economic development and population growth. Pressure-driven membrane processes such as microfiltration (MF), ultrafiltration (UF), nanofiltration (NF), and reverse osmosis (RO) are currently considered as the most commonly-used processes for water treatment [1,2].

Polymeric membranes, despite being the most commonly-used in water treatment, have some concerns associated with them, such as: hydrophobicity, fouling, stability (mechanical, thermal, and chemical), etc. hence, the preparation of novel membranes with enhanced properties and performance is of vital significance for practical applications of membrane separation processes for desalination and wastewater treatment [3,4]. Such novel membranes must be engineered to deliver the specific water treatment needs by altering their physicochemical and structural properties, such as: porosity, hydrophilicity, chemical, mechanical, and thermal properties in addition to the incorporation of some other important features such as: adsorption, conductive or antibacterial capabilities [5]. In addition to high selectivity and permeate flux, water treatment membranes for industrial processes require good mechanical and thermal properties, stability, and durability. In pressure-driven membrane processes, the mechanical properties of the polymeric membranes are crucial as at high operating pressure, which, for example for RO may reach up to 50–60 bars, the membranes undergo physical compaction which results in an irreversible drop in the permeate water flux. Moreover, polymeric membranes characterized with low tensile strength values are vulnerable to failure due to operational stresses such as the high operating pressures [6,7,8].

Recently, the incorporation of nanomaterials into polymeric matrices for the sake of preparation of advanced membranes with enhanced properties has been widely reported in literature [3,9]. Nano-fillers are introduced into polymers to synthesize porous composite membranes with improved thermal and mechanical performance for desalination and water treatment, [10,11,12,13]. CNTs is an example of carbon-based nanomaterials which are widely added to synthesize advanced hybrid inorganic-polymer membranes with enhanced properties due to their outstanding thermal, chemical, and mechanical stability in addition to their exceptional antibacterial and conductive behavior [14,15,16,17,18]. CNTs have received considerable attention from academia and industry over the last twenty years. CNTs are carbon atoms sheets which are rolled like a cylinder to make smooth hollow tubes. There are two types of CNTs which have been described in literature; these are single-walled carbon nanotubes and multi-walled carbon nanotubes [9]. CNTs are expected to be excellent reinforcement for polymer membranes, improving the permeability as well as mechanical properties [19]. So far most of the reported work on incorporation of CNTs into polymer membranes has focused on evaluating the separation and antifouling properties of composite membranes [20,21,22]. Marand et al. [23] investigated the effect of CNTs incorporation on the performance of PS membranes for gas separation application. They reported the increase in the permeabilities and diffusivities of the membranes along with the increasing in the loading of CNTs. Limited studies, however, emphasized on the mechanical properties of hybrid CNT-polymeric membranes and the effect of the CNTs features (such as fabrication technique, aspect ratio, etc.) on the performance of the membranes. Majeed et al. [24] reported an increase of mechanical strength of a polyacrylonitrile (PAN)/multi walled carbon nanotubes (MWCNTs) UF membranes with increasing MWCNTs loading up to 2 wt. % at room temperature as a result of the reduction in the porosity and enhancement in the interaction between the PAN matrix and MWCNTs. Maphutha et al. [25] reported that the mechanical properties of a polysulphone (PS)/CNTs membrane first increased with increasing CNT loading and then decreased after a threshold CNTs concentration of 7.5 wt. %. The decreased mechanical properties of the membranes were attributed to the agglomeration of CNTs creating bundles at high CNT contents.

The purpose of this work is to provide a new insight into the microstructure properties relationship of PS/CNTs nanocomposite membranes, as a function of CNTs’ content, aspect ratio, and surface functional groups. To this end, the surface property of the membranes was studied by water contact angle measurement. The water flux of the membranes was investigated and correlated to their porosity. The mechanical behaviors of the PS/CNTs nanocomposite membranes and their macroscopic mechanical responses were evaluated and correlated with the membrane′s morphology at different temperatures as that experienced in water treatment applications.

## 2. Experimental

### 2.1. Materials and Processing

In this work, two kinds of CNTs, namely commercial and laboratory-made (produced) CNTs with different aspect ratios, were used for the preparation of PS/CNT nanocomposite membranes. The commercial CNTs were obtained from the Chengdu Organic Chemicals Co., Ltd. (Chengdu, China) and had lengths of 1 µm to 10 µm and outer diameters of 10 nm to 20 nm [26]. The produced CNTs with high-purity, high-quality, high surface area, and high aspect ratio were synthesized by using a vertical atomizer chemical vapor deposition reactor (VA-CVD) via a gas phase. The main advantages of this VA-CVD are its absolute ability to mass-produce nanotube materials and the controllable growth of CNTs [27]. The produced CNTs had diameters ranging from 20 to 50 nm and lengths ranging from 300 to 500 microns [26]. It should be noted that due to acidic treatment commercial CNTs contain carboxylic groups on their surface. The main properties of both commercial and produced CNTs are summarized in Table 1.

PS nanocomposite membranes incorporated with different CNTs content of 0.1–1.0 wt. % were casted via a phase inversion method. The corresponding amount of CNTs was sonicated in dimethylacetamide for 1 h in order to allow for the complete dispersion of CNTs in the solvent. The surfactant, poly(vinylpyrrolidone) with a molecular weight of 50 kDa, was added prior to stirring for 30 min. After that, PS was added and the dope solution was mixed for 24 h at 60 °C. The casting solution was characterized with dark color, homogeneous texture and with the absence of obvious macroscopic agglomeration. This solution was then degassed for half an hour and left under vacuum for 24 h to remove any air bubbles trapped in the solution. Eventually, the solutions were cast uniformly onto a cleaned smooth glass plate at room temperature using a casting knife with a 200 mm gap height and a casting speed of 2.5 cm/s. After casting, the glass plate, upon which the membrane film was cast, was kept for half a minute before being immersed into the non-solvent coagulation bath with deionized water (DW) at 25 °C. After some time, the membrane film was observed to peel off from the glass plate. The prepared membranes, which have an average thickness of about 100 µm, were washed with DW. The cast membranes were immersed in DW bottles for 24 h at room temperature to remove any remaining solvent. Two different sets of PS membranes embedded with commercial and produced CNTs at the same CNT loading, were prepared at the same casting conditions. In the following, PS/CNTs nanocomposite membranes were denoted as PS/x wt. % CCNTs and PS/x wt. % PCNTs for the membranes with commercial and produced CNTs, respectively, where x is a CNT weight content, wt. %.

### 2.2. Characterization

Powder X-ray diffraction (XRD) patterns using Rigaku MiniFlex-600 X-ray (Ridaku, Austin, TX, USA) diffractometer with Cu Kα radiation λ = 1.54 Å at a rate of 0.4% over Bragg angles ranging from 10–90 degrees was used for the crystalline dimensions and phase identification of CNT. The operating current and voltage were maintained at 15 mA and 40 kV, respectively.

Transmission electron microscopy (TEM) (CM12, Philips, Eindhoven, The Netherlands) was used to carry out the structural and morphological analysis of CNTs.

Water contact angle (WCA) measurements were characterized following the sessile drop technique with Ramé-hart Model 200 Standard Contact Angle Goniometer (Ramé-hart instrument Co., Succasunna, NJ, USA), using high purity water. The size of the water drops was 2.0 μL. Twenty-five measurements per membrane sample were taken. This was conducted by placing 5 water droplets at 5 different locations in the sample. After that, the contact angle of every water drop was measured 5 times which results in 25 measurements per membrane sample.

In order to evaluate the water flux of the prepared membranes, a Sterlitech HP 4750 (Sterlitech, Kent, WA, USA) dead-end membrane cell was used. The effective membrane area was about 14.6 cm^2^. The membrane permeate flux (*J*) was estimated by measuring the time required to collect some permeate volume, which had been passed through the membrane, using the Equation (1):

(1) $$J=\frac{V}{At}$$

where *J* stands for flux (L/m^2^h), *V* is permeate volume (L), *A* is the effective membrane area (m^2^), and *t* is the time required to collect the permeate volume (h).

The overall porosity of membranes were determined as described by Zheng et al. [28]. The membranes were immersed in water for one day, then removed from water and then wiped off carefully with filter paper to take away any extra water before weighting. Next, the membranes were dried in a 60 °C oven over night and eventually weighted. Equation (2) was used to figure out the overall porosity:

(2) $$P=\frac{W_{1}-W_{0}}{\rho A_{s}h}$$

where *P* is the overall membrane porosity (%), *W*_1_ and *W*_0_ are the wet and dry weights of the membranes (g), respectively, *ρ* is the water density at room temperature (g/cm^3^), *A_s_* is the membrane surface area (cm^2^) and *h* is the thickness of the membrane (mm).

The morphology of bare PS and PS/CNTs membranes was studied using two techniques; namely: (1) Field Emission Scanning Electron microscope (FE-SEM, MIRA3 TESCAN) with 15 kV accelerating electron voltage and (2) atomic force microscopy (AFM). Prior to SEM analysis, the membrane samples were gold-coated with 5 nm layer using Ion Sputter Q 150R S (Quorum Technologies, Lewes, UK). On the other hand, the morphology characterization using AFM was evaluated using a Dimension Icon model AFM with NanoScope V Controller (Bruker AXS, Fitchburg, WI, USA) which employed PeakForce mode. All AFM measurements were conducted using NSG30 silicon tapping mode probes (NT-MDT, Moscow, Russia, nominal spring constant = 40 N/m, nominal tip radius of curvature = 5 nm) at room temperature.

MTS Insight universal test system with a loading cell of 1 KN was used to figure out the uniaxial tensile behavior of the prepared membranes under different testing conditions. Wet specimens with a width of 10 mm and a length of 90 mm were cut from the cast membranes. Tensile testing was conducted at three different temperatures; namely: 24 °C (room temperature), 40 °C, and 60 °C which is similar to the temperature experienced in water treatment applications. To ensure the uniformity of the temperature of the membrane specimen, at every temperature change, the empty chamber was initially heated for 30 min. After that, the membrane specimen was inserted in the chamber for 5 min to accommodate for thermal equilibration before carrying out the testing. The Young’s modulus, yield stress, and the elongation at fracture of the materials were assessed at a crosshead speed of 5 mm/min according to ISO 527-3. Since the tensile specimens have a gauge length of 50 mm, tensile procedures were conducted at an initial strain rate of 0.17/s. Eight tests were performed for every material composition to determine the average values for every mechanical parameter.

## 3. Results and Discussion

### 3.1. CNTs Characterization

**Figure 1:**
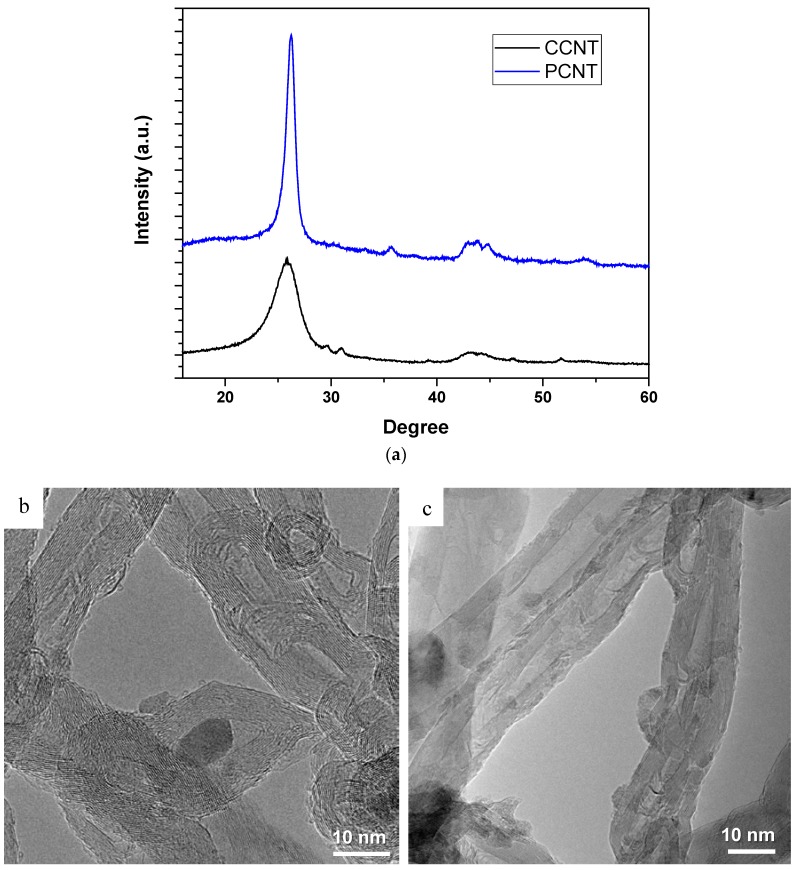

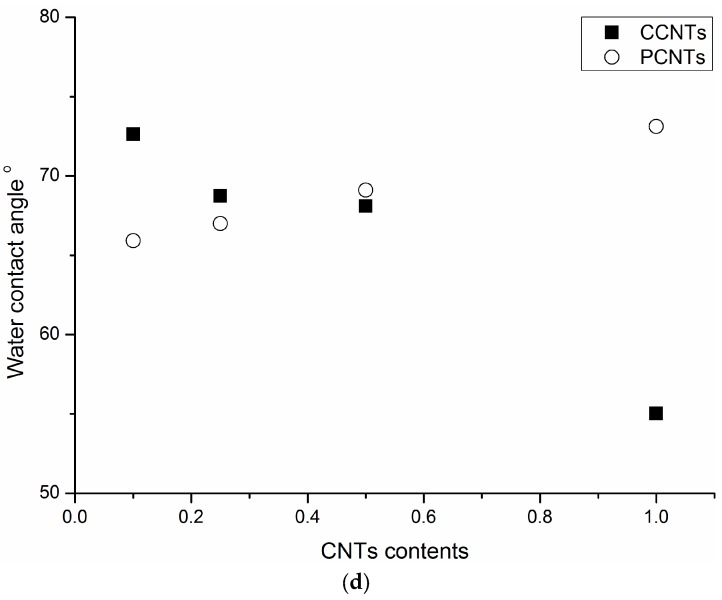
XRD characterization of PCNT and CCNT (**a**), TEM images of CCNT (**b**) and PCNT (**c**), and Water contact angle of PS/CNTs nanocomposite membranes as a function of CNTs content (**d**).

Figure 1a shows the XRD pattern for both commercial and CNTs prepared in the lab. The CNT distinctive peak appears at the angle (2θ) of 25.5° which can be indexed as the C(002) representing the hexagonal graphite structure. Other available characteristic diffraction peaks of CNTs or graphite in general appears at 2θ of about 43°, 45°, and 77° and they are indexed as C(100), C(101), and C(110) diffractions of graphite, respectively. The peaks indexed at (002), (100), (101) represent hexagonal structure of graphite but presence of 002 peak in the CNT XRD data, is revealing the multi-walled nature of the CNTs. Based on the XRD result, the CNTs produced in the lab showed good crystallinity, highly ordered and uniform, and extremely pure compared to the commercially purchased CNTs as indicated by the C(002) peak in the XRD data. These have been confirmed by TEM images in Figure 1b,c.

In order to characterize the morphology and structure of the produced and commercial CNTs, TEM analysis was conducted and depicted in Figure 1b,c which shows the TEM images of produced and commercial CNTs (PCNTs and CCNTs, respectively). As it is sown in Figure 1b,c, the CCNTs are highly entangled and non-uniform with presence of some major defects on their wall. The average size of CCNTs seen from TEM image in Figure 1b is in range of 20–30 nm. On the other hand, Figure 1c shows the TEM image of PCNTs which are highly uniform, highly ordered, and crystalline and no major deformation or defect on their wall can be seen. The average size of PCNT estimated from TEM image is in the range of 10–20 nm.

### 3.2. Membrane Characterization

The surface hydrophilicity of the PS/CNTs nanocomposite membranes was found to be affected by the addition of CNTs. Figure 1d displays water contact angle (WCA) of PS/CNTs nanocomposite membranes as a function of CNTs content.

The WAC for PS/CCNTs nanocomposite membranes sharply decreased with increasing CCNTs content: from 73° to 53° for PS/CCNTs nanocomposite membranes with 0.1 and 1.0 wt. % of CCNTs, respectively, showing a high degree of correlation (R^2^ = 0.954). As was mentioned before, due to acidic treatment, commercial CNTs contain carboxylic groups on their surface. During the membrane casting via the phase inversion process, the acidic-treated hydrophilic CCNTs migrated to the membrane surface, making the membrane surface more hydrophilic. In comparison, the WCA for PS/PCNTs nanocomposite membranes increased with increasing PCNTs content: from 66° to 72° for PS/PCNTs nanocomposite membranes with 0.1 and 1.0 wt. % of PCNTs, respectively (R^2^ = 1.000), suggesting the hydrophobic properties of the PCNTs.

**Figure 2:**
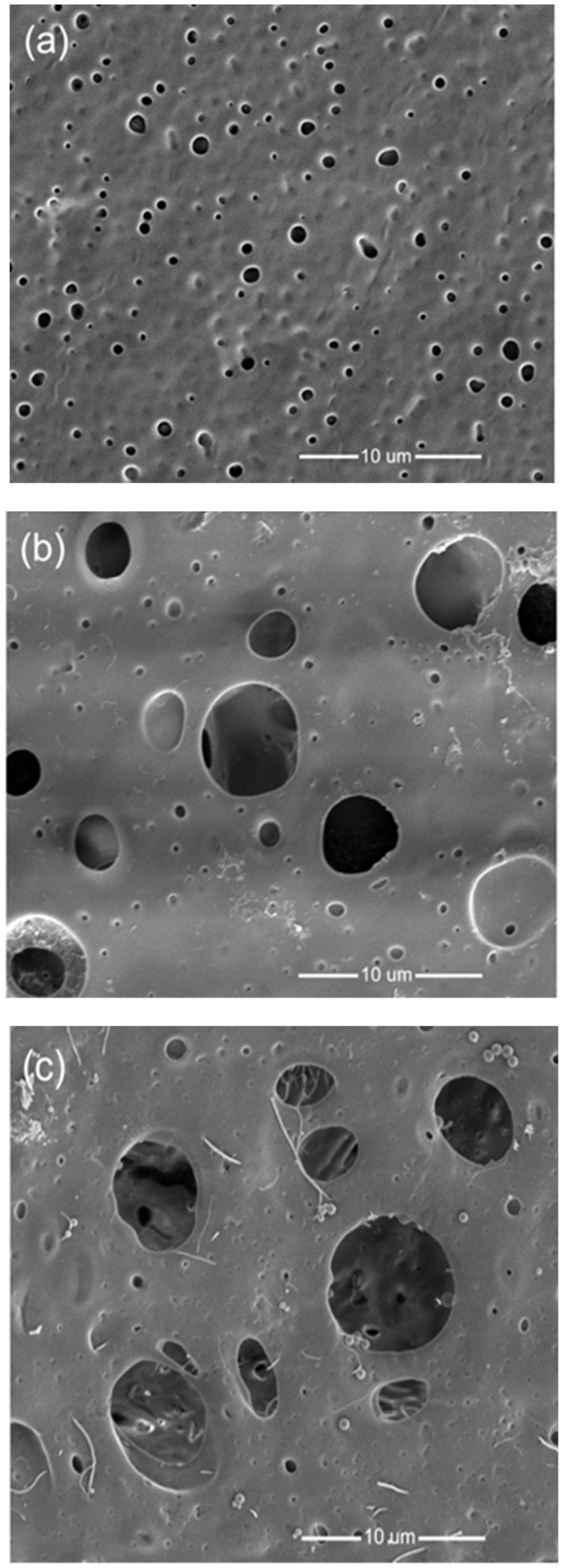
SEM micrographs of (**a**) pristine PS, (**b**) PS/ 1.0 wt. % CCNT, and (**c**) PS/ 1.0 wt. % PCNT membranes.

SEM micrographs of the pristine PS membranes and PS/CNT nanocomposite membranes with 1.0 wt. % of CCNT or PCNT are exhibited in Figure 2a–c, respectively. The SEM images for other membranes with lower CNT content have similar trends. In Figure 2, the pore size of pristine membranes (Figure 2a) was smaller than that of PS/1.0 wt. % CCNTs (Figure 2b) and PS/1.0 wt. % PCNTs (Figure 2c) nanocomposite membranes. Previously, it was observed that the introduction of CNTs to the casting solution led to a faster solvent and non-solvent phase exchange (due to the thermodynamic immiscibility) and hence resulted in the formation of a more pronounced porous membrane structure [29].

Both CCNTs and PCNTs were well dispersed and distributed within the PS matrix. The PCNTs observed in Figure 2c were much longer than the CCNTs observed in Figure 2b. In addition, both CCNTs and PCNTs were well incorporated into the PS membrane matrix.

**Figure 3:**
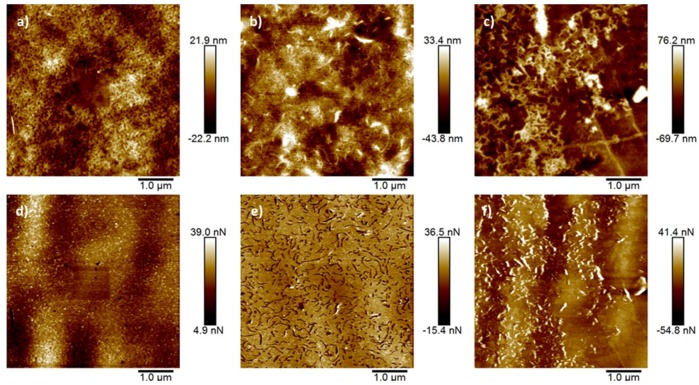
AFM images of PS membranes: (**a**) height image of pristine PS (root mean square roughness (Rq) for this image = 6.47 nm); (**b**) PS with commercial CNTs (Rq = 12.9 nm); (**c**) PS with produced CNTs (Rq = 25.6 nm); (**d**) adhesion map of pristine PS obtained simultaneously with image (**a**); (**e**) adhesion map of PS with commercial CNTS corresponding to (**b**); (**f**) adhesion map of PS with produced CNTs corresponding to (**c**).

The surface morphology, obtained by AFM, of pristine PS membranes, PS/PCNTs, and PS/CCNTs membranes is presented in Figure 3 alongside adhesion maps collected simultaneously with topographic images. Root mean squared roughness values for each membrane is indicated in the figure caption. As is apparent, the presence of 1.0 wt. % CNTs, both commercial and produced, led to an increased surface roughness when compared with the pristine membrane surface. Adhesion maps of the 1.0 wt. % CCNT containing membrane surfaces (Figure 3e) show a strong contrast between the membranes and the CNTs, with the CNTs demonstrating lower measured adhesion forces (darker) than the surrounding membrane. This is what would be expected with hydrophilic CCNTs imaged with a hydrophobic AFM probe (WCA measurements on the surface of the AFM probes (0.5 μL drop size) showed a mean contact angle of 133°). Inversely, for the PCNTs the contrast was much less marked, but showed greater adhesion forces measured between the AFM probe tip and the CNTs compared with the surrounding PS membrane matrix, which fits with the PCNTs being hydrophobic. Modification of the surface by addition of PCNTs and CCNTs in this way is in accordance with and explains the observed changes in WCA seen with the contact angle measurements.

**Figure 4:**
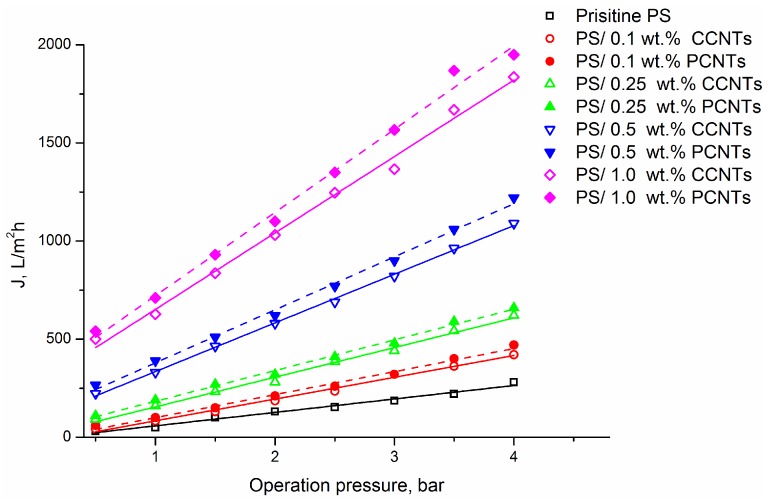
Water fluxes of PS and PS/CNTs nanocomposite membranes at different CNTs loading versus operating pressure.

The pure water fluxes of PS membranes fabricated at different concentrations of CCNTs or PCNTs in the casting solutions are presented in Figure 4. The PS/CNT nanocomposite membranes exhibited considerably increased permeate fluxes (up to 20 times) compared to the pristine PS membrane and these fluxes are typical for wide-pored UF and MF membranes. As seen in Figure 4, the flux values increased with CNT content in the casting solution. These findings can be related to the porosity data of the prepared membranes. As seen in Table 2, the total porosity of the nanocomposite membranes increased with increasing CNT content that resulted in higher water flux values. It should be also noted that at the same CNT content, water fluxes for PS membranes embedded with PCNTs are higher than those for the samples with incorporated CCNTs.

These results were found to agree with what was reported by Marand et al. [23] who investigated the effect of CNTs incorporation on the performance of PS membranes for gas separation application. They reported the increase in the permeabilities and diffusivities of the membranes along with the increasing in the loading of CNTs.

**Figure 5:**
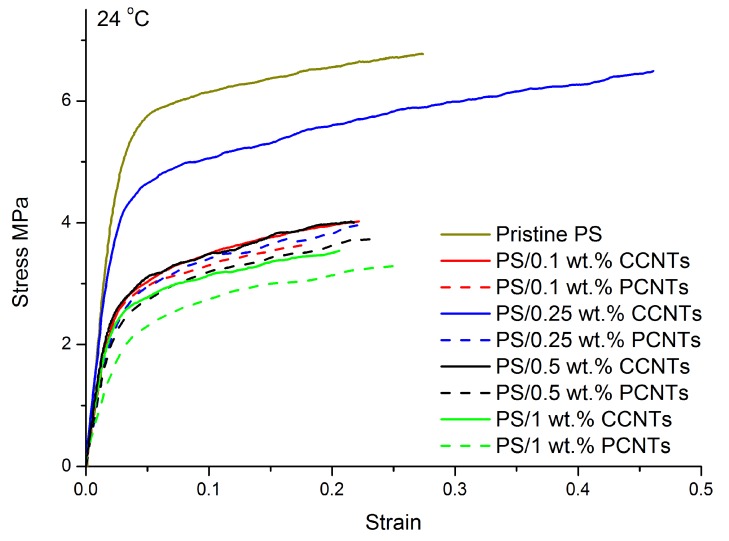
Engineering stress–strain curves of PS nanocomposite membranes with different contents of CCNTs or PCNTs at room temperature and at an initial strain rate of 0.17/s.

**Figure 6:**
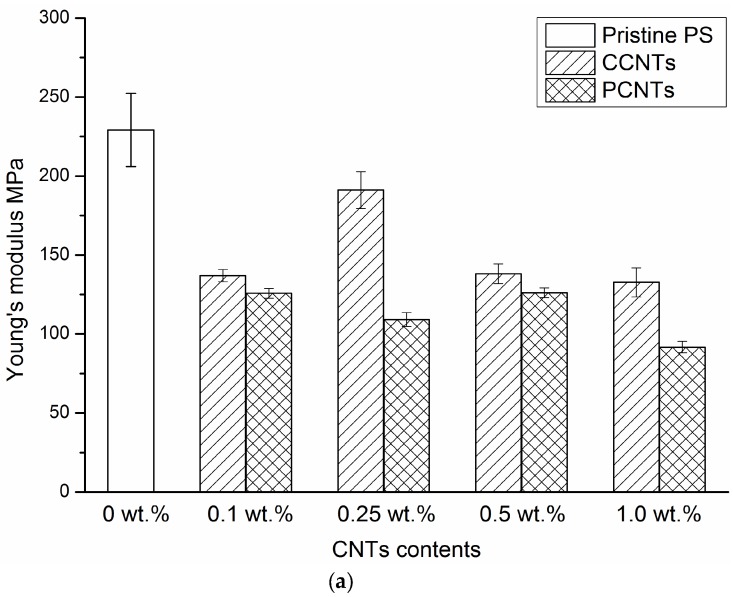

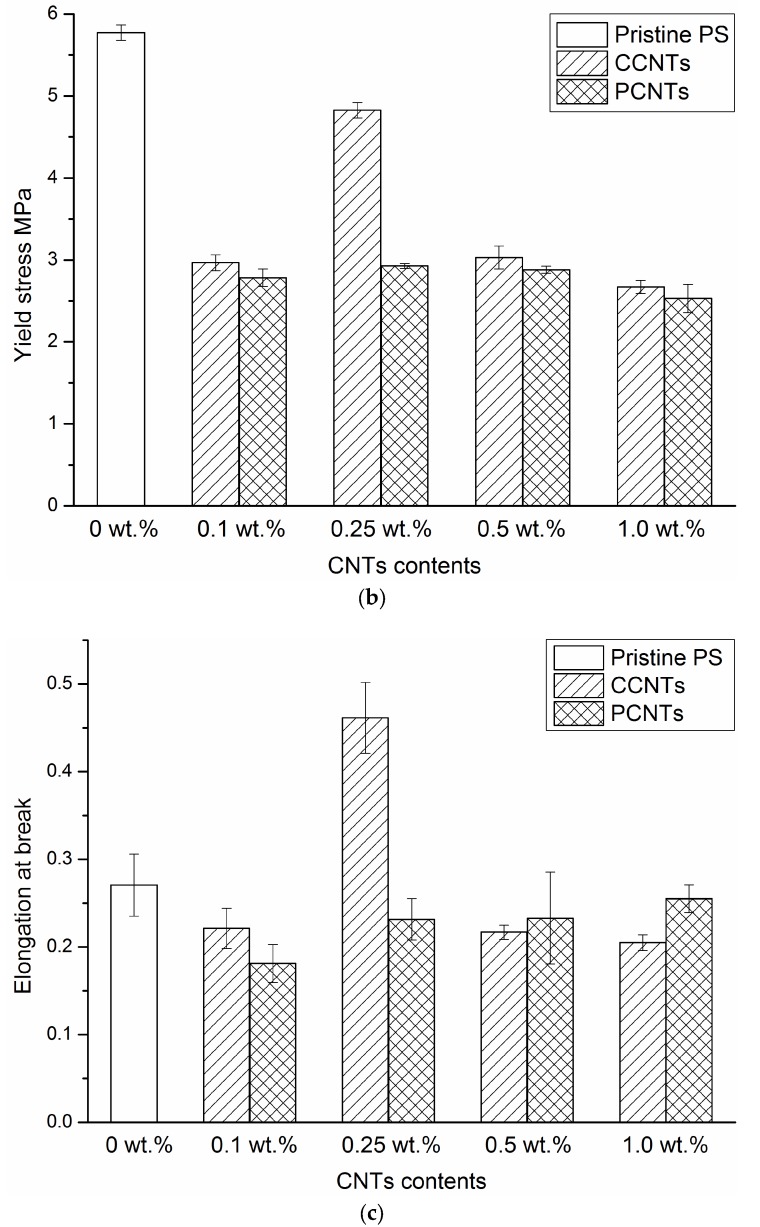
Young’s modulus (**a**), yield stress (**b**), and elongation at break (**c**) of PS nanocomposite membranes with different contents of CCNTs and PCNTs at room temperature at an initial strain rate of 0.17/s.

Figure 5a shows the engineering stress–strain curves of PS nanocomposite membranes with different content of CCNTs or PCNTs at room temperature and at an initial strain rate of 0.17/s. The extracted mechanical parameters such as Young’s modulus, yield stress, and elongation at fracture are shown in Figure 6a–c and summarized in Table 3. In Figure 5, the tensile behaviors of all the PS/CNTs nanocomposite membranes exhibited three stages: linear elasticity, nonlinear transition to yield, and post-yield strain hardening. Pristine PS membrane had the highest tensile strength, as characterized by a Young’s modulus of 229 MPa, a yield stress of 5.8 MPa and a fracture strain of 0.27. With the addition of 0.1 wt. % CCNTs or PCNTs, the mechanical properties of the PS/CNTs nanocomposite membranes significantly decreased. For example, the Young’s modulus decreased from 229 MPa for pristine PS membrane to 137 MPa for PS/0.1 wt. % CCNTs membrane, representing a decrease of 40.2%. The yield stress decreased from 5.8 MPa for pristine PS membrane to 3.0 MPa for PS/0.1 wt. % CCNTs membrane, representing a decrease of 48.3%. However, with the addition of 0.25 wt. % CCNTs, the tensile properties of the membrane increased compared with those of PS/0.1 wt. % CCNT nanocomposite membrane. The Young’s modulus of PS/0.25 wt. % CCNT membrane had a decrease of 16.6% but its elongation at fracture had a significant increase of 70.4% compared with the pristine PS membrane. With increasing CCNT or PCNT content (0.5 wt. % and 1.0 wt. %), the mechanical properties of the membranes decreased again compared with those of PS/0.25 wt. % CCNT membrane. Note that the Young’s modulus and yield stress of the membranes with CCNT were slightly higher than those of the membranes with the PCNTs for a given CNT content, except for the membranes with 0.25 wt. % CNTs. More specifically, the magnitude of the mechanical properties of the PS/0.25 wt. % CCNTs were much higher than those of the PS/0.25 wt. % PCNT nanocomposite membranes.

The mechanical properties of porous membranes depend to a great extent on the average pore size, porosity, and pore size distribution of the membranes. For porous composite membranes with identical matrix and fillers, processing conditions and filler content, their mechanical properties are anticipated to be better for membranes with smaller pore size and porosity [8]. In Figure 5, the higher tensile behavior of the pristine PS membrane is mainly due to its lower porosity. The exceptionally high aspect ratio, in addition to the high strength and stiffness, make CNTs a potential candidate as a reinforcement for polymer materials. Therefore, the use of CNTs was expected to increase the mechanical properties of the PS membranes. In the present study, PS membranes incorporated with the CCNTs or PCNTs had lower mechanical properties compared with the pristine PS membrane, which is probably due to the higher porosity of the PS/CCNTs and PS/PCNTs nanocomposite membranes (see Table 2). This indicates that the tensile behavior of the PS/CNTs nanocomposite membranes is based on the competition between strengthening of membranes due to CNT reinforcement and softening due to their porous microstructure, with the effect of average porosity seeming to be the more dominant factor affecting the tensile properties for the loading ratios used in this study. In addition, the relatively lower Young’s moduli and yield stresses for PS/1.0 wt. % CCNTs and PS/1.0 wt. % PCNTs compared with other PS/CNTs membranes are most likely due to the stress concentration induced by CNT aggregation at high CNT content. The improvement of ductility for PS/0.25 wt. % CCNT membrane is probably due to the good dispersion and distribution of the CCNTs within the PS matrix, and the good interfacial adhesion between the CCNTs and the PS matrix, which allowed better stress transfer from the matrix to the CNTs delaying the rupture of the membranes. Even though the PS nanocomposite membranes containing high aspect ratio PCNTs were expected to have higher mechanical behaviors than the membranes filled with the CCNTs [30], the higher porosity of the PS/PCNT membranes compared with that of the PS/CCNTs for a given CNT content resulted in lower Young’s moduli and yield stresses. The higher porosity of the PS/PCNT membranes compared with PS/CCNT ones is most likely due to enhanced phase inversion during the membrane casting. Moreover, enhanced compatibility of the CCNTs with PS may be another reason to explain the higher mechanical properties of the PS/CCNT nanocomposite membranes for a given CNT content. More specifically, the presence of carboxylic groups on the surface of CCNTs offered multiple sites for hydrogen bonding between these groups and sulfonic groups of PS improving the membranes mechanical properties. The results show, in our case, the CNTs’ aspect ratio had a less pronounced effect on mechanical features of the membranes than compatibility of CNTs with PS matrix and the membranes’ porosity.

**Figure 7:**
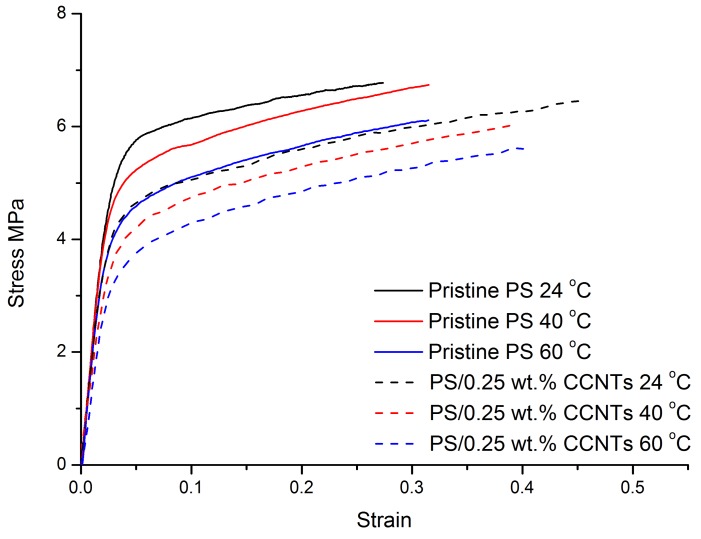
Engineering stress–strain curves of PS nanocomposite membranes with different contents of CCNTs or PCNTs at different temperatures and at an initial strain rate of 0.17/s.

Engineering stress–strain curves of pristine PS membranes and PS/0.25 wt. % CCNT nanocomposite membranes at different temperatures and at the initial strain rate of 0.17/s are shown in Figure 7. In these curves, the effect of temperature can be observed for pristine PS membranes and PS/0.25 wt. % CCNTs nanocomposite membranes. It can be seen that the Young’s modulus and yield stress decreased with increasing temperatures. However, the impact of temperature on the post-yield behaviors and on the fracture strains of the pristine PS membranes and PS/0.25 wt. % CCNT nanocomposite membranes was limited. All the curves show similar post-yield strain-hardening slope. The fracture strain for pristine PS membranes at 40 °C and 60 °C were slightly higher than that at 24 °C, whereas the fracture strain for PS/0.25 wt. % CCNT nanocomposite membranes at 40 °C and 60 °C were slightly lower than that at 24 °C.

When the temperature rises, the distance between the polymer structural units increases due to thermal agitation and in consequence, interaction forces decrease, which implies a degradation of mechanical properties. Therefore, the Young’s modulus and yield stress of pristine PS membranes and PS/0.25 wt. % CCNT nanocomposite membranes decreased for higher temperatures. In Figure 6a, the less changed elongation at fracture with increasing temperatures for both membranes is probably due to the high glass transition temperature of PS which is around 185 °C. In our investigated range, the maximum temperature of 60 °C was still considerably less than the glass transition temperature of PS. Therefore, the increased temperature had limited effect on the motion of polymer chains, resulting in similar fracture strains for the membranes at higher temperatures. This reason may also be used to explain the less changed post-yield strain-hardening slopes for both membranes with increasing temperatures. In addition, drying of the membranes during heating by continuous air circulation in the environmental chamber is another likely reason for the less changed fracture strain with increasing temperature for both membranes. In order to measure the fracture strain of dried membranes, tensile tests were also conducted at room temperature under the same crosshead speed. It was found that the elongation at fracture of dried pristine PS membranes was 0.19, which was much lower than the fracture strain of 0.27 measured for wet membranes under the same testing conditions. Therefore, drying of the membranes by the environmental chamber had an important effect on the fracture strain of the membranes in our investigated range. Note that temperature’s effects on the mechanical behaviors of PS nanocomposite membranes with other CCNT or PCNT contents are not plotted here because they have similar results than the pristine PS membranes and PS/ 0.25 wt. % CCNT nanocomposite membranes.

## 4. Conclusions

In this work, novel PS/CNTs nanocomposite membranes incroporated with CCNTs or PCNTs were prepared using a phase inversion method. The results indicated that the incorporation of CNTs into the polymer matrix significantly enhances the performance of the fabricated nanocomposite membranes and must be controlled carefully to achieve the optimum results. It was observed that changes related to the CNT (such as: CNT loading in the dope solutions, aspect ratio, and functionality of CNTs) have significantly changed the membrane properties and performance, such as: porosity, water flux, and mechanical and surface properties of the prepared PS/CNT membranes. The permeate water flux of the fabricated membranes has been observed to increase severely (up to 20 times), whereas the yield stress and Young’s modulus have dropped along with the increase in the CNT loading as a result of higher porosity in the prepared membranes. It was also observed that the mechanical properties of the membranes were not only influenced by CNTs’ loading and aspect ratio but also the affinity of CNTs to the polymeric matrix. The elongation at fracture for PS/0.25 wt. % CCNT membrane was much higher than for pristine PS membrane, obviously, due to enhanced compatibility of commercial CNT with PS via hydrogen bonding. It was found that a more pronounced effect on the membrane’s mechanical properties was observed due to the compatibility of CNTs with PS matrix when compared to other factors, such as changes in the aspect ratio of CNT.

The obtained results show that complex interplay of various factors such as: loading of CNT in the dope solutions, aspect ratio, and functionality of CNT should be carefully considered for the preparation of PS/CNTs nanocomposite membranes with enhanced mechanical properties and performance. This means that more thorough work must be conducted in order to have a better understanding of the relationship between the membrane structure and its properties. This includes the failure mechanisms at different temperatures which in turn would offer a solid scientific basis for rational engineering of produced functionalized CNTs of different aspect ratios and for manufacturing of composite PS/CNTs membranes with optimized performance and mechanical properties.

## Figures and Tables

**Table 1:** Main properties of commercial and produced CNTs.

CNTs’ Property	Commercial CNTs	Produced CNTs
Type	Multiwall	Extra-long multiwall
Production technique	CVD	CVD
Diameter (nm)	10–20	20–50
Length (µm)	1–10	200
Aspect ratio	100–500	4000–20,000

**Table 2:** Total porosity of PS/CNTs nanocomposite membranes.

Membrane									
CNTs Type	-	CCNTs	PCNTs	CCNTs	PCNTs	CCNTs	PCNTs	CCNTs	PCNTs
Loading (wt. %)	0	0.1	0.1	0.25	0.25	0.5	0.5	1.0	1.0
Porosity, %	30.4 ± 0.7	32.1 ± 0.8	33.5 ± 0.6	35.4 ± 0.5	38.2 ± 0.9	43.3 ± 0.8	44.7 ± 0.8	50.4 ± 0.6	51.6 ± 0.7

**Table 3:** Mechanical behaviors of PS nanocomposite membranes with different contents of CCNTs or PCNTs at room temperature and at an initial strain rate of 0.17/s.

Membranes	Young’s Modulus (MPa)	Yield Stress (MPa)	Elongation at Fracture
Pristine PS	229.2 ± 23.2	5.8 ± 0.1	0.27 ± 0.04
PS/0.1 wt. % CCNTs	136.9 ± 3.9	3.0 ± 0.1	0.22 ± 0.02
PS/0.1 wt. % PCNTs	125.8 ± 3.2	2.8 ± 0.1	0.18 ± 0.02
PS/0.25 wt. % CCNTs	191.2 ± 11.7	4.8 ± 0.1	0.46 ± 0.04
PS/0.25 wt. % PCNTs	109.2 ± 4.4	2.9 ± 0.0	0.23 ± 0.02
PS/0.5 wt. % CCNTs	138.2 ± 6.3	3.0 ± 0.1	0.22 ± 0.01
PS/0.5 wt. % PCNTs	126.1 ± 3.2	2.9 ± 0.0	0.23 ± 0.05
PS/1.0 wt. % CCNTs	132.7 ± 9.2	2.7 ± 0.1	0.21 ± 0.01
PS/1.0 wt. % PCNTs	91.7 ± 3.6	2.5 ± 0.2	0.26 ± 0.02
